# Yeast response and tolerance to benzoic acid involves the Gcn4- and Stp1-regulated multidrug/multixenobiotic resistance transporter Tpo1

**DOI:** 10.1007/s00253-017-8277-6

**Published:** 2017-04-13

**Authors:** Cláudia P. Godinho, Nuno P. Mira, Tânia R. Cabrito, Miguel C. Teixeira, Kaur Alasoo, Joana F. Guerreiro, Isabel Sá-Correia

**Affiliations:** Institute for Bioengineering and Biosciences (iBB), Department of Bioengineering, Instituto Superior Técnico, Universidade de Lisboa, Avenida Rovisco Pais, 1049-001 Lisbon, Portugal

**Keywords:** Multidrug/multixenobiotic resistance transporters, Weak acid food preservatives, Adaptation and tolerance to benzoic acid, Polyamines, *TPO1*/Tpo1, *Saccharomyces cerevisiae*

## Abstract

The action of benzoic acid in the food and beverage industries is compromised by the ability of spoilage yeasts to cope with this food preservative. Benzoic acid occurs naturally in many plants and is an intermediate compound in the biosynthesis of many secondary metabolites. The understanding of the mechanisms underlying the response and resistance to benzoic acid stress in the eukaryotic model yeast is thus crucial to design more suitable strategies to deal with this toxic lipophilic weak acid. In this study, the *Saccharomyces cerevisiae* multidrug transporter Tpo1 was demonstrated to confer resistance to benzoic acid. *TPO1* transcript levels were shown to be up-regulated in yeast cells suddenly exposed to this stress agent. This up-regulation is under the control of the Gcn4 and Stp1 transcription factors, involved in the response to amino acid availability, but not under the regulation of the multidrug resistance transcription factors Pdr1 and Pdr3 that have binding sites in *TPO1* promoter region. Benzoic acid stress was further shown to affect the intracellular pool of amino acids and polyamines. The observed decrease in the concentration of these nitrogenous compounds, registered upon benzoic acid stress exposure, was not found to be dependent on Tpo1, although the limitation of yeast cells on nitrogenous compounds was found to activate Tpo1 expression. Altogether, the results described in this study suggest that Tpo1 is one of the key players standing in the crossroad between benzoic acid stress response and tolerance and the control of the intracellular concentration of nitrogenous compounds. Also, results can be useful to guide the design of more efficient preservation strategies and the biotechnological synthesis of benzoic acid or benzoic acid-derived compounds.

**Electronic supplementary material:**

The online version of this article (doi:10.1007/s00253-017-8277-6) contains supplementary material, which is available to authorized users.

## Introduction

Benzoic acid is a lipophilic weak acid that occurs naturally in many plants, is largely used in the preservation of foods and beverages and is an intermediate compound in the biosynthesis of many secondary metabolites. Uncovering the complexity of cellular responses to stress induced by benzoic acid in the experimental eukaryotic model *Saccharomyces cerevisiae* might be instrumental to improve food preservation action and microbial performance in biotechnological processes. In particular, the identification of candidate genes and signalling pathways involved in the response and resistance to this stress is essential to find targets for genetic engineering to increase stress robustness for biotechnological processes or to guide preservation strategies (Dos Santos and Sá-Correia 2015; Mira et al. 2010b; Teixeira et al. 2011b). Multidrug/multixenobiotic resistance (MDR/MXR) is many times the result of the action of MDR/MXR transporters found at the membranes of all living cells (Sá-Correia et al. 2009; Teixeira et al. 2011a). Therefore, our laboratory has dedicated research efforts to the study of the biological role and regulation of drug/xenobiotic pumps of the major facilitator superfamily (MFS) and the ATP-binding cassette (ABC) superfamily, and the link between their physiological role and the MDR/MXR phenomenon in yeast (Sá-Correia et al. 2009). The *S. cerevisiae* plasma membrane drug:H^+^ antiporter (DHA) Tpo1, a MDR/MXR transporter of the MFS, has been found to mediate tolerance of this yeast species to a high number of cytotoxic compounds including the metal ions cadmium and aluminium (Cabrito et al. 2009), the antimalarial drugs quinidine and artesunate (Alenquer et al. 2006; do Valle Matta et al. 2001), the immunosuppressant mycophenolic acid (Desmoucelles et al. 2002), the herbicides 2,4-dichlorophenoxyacetic acid (2,4-D) and barban (Cabrito et al. 2009; Teixeira and Sá-Correia 2002), the anticancer agent bleomycin (Berra et al. 2014; Hillenmeyer et al. 2008), the antifungals nodoconazole and mancozeb (Dias et al. 2010; Hillenmeyer et al. 2008), the nonsteroidal anti-inflammatory drug diclofenac (Mima et al. 2007) and the weak acids acetic, propionic, decanoic and octanoic acids (Borrull et al. 2015; Legras et al. 2010; Mira et al. 2009). The apparent promiscuity of Tpo1 and other yeast MFS-MDR transporters in conferring protection to a wide range of structurally unrelated xenobiotic compounds has been questioning the idea that these transporters contribute to MDR by directly mediating the extrusion of the drugs (Dos Santos et al. 2014; Mira et al. 2010a; Sá-Correia et al. 2009). Within this line of thought, evidences have been obtained showing that the beneficial effect of some yeast MDR pumps in conferring drug resistance is indirect and results from their effect in the transport of a given physiological substrate whose partition ends up contributing to reduce the internal accumulation of drugs or to counteract their deleterious effects (Cabrito et al. 2011; Sá-Correia et al. 2009; Teixeira et al. 2011a; Vargas et al. 2007). A paradigmatic example has been Qdr2, whose protective effect against quinidine action was correlated with its role in K^+^ uptake (Vargas et al. 2007). Tpo1 has been found to be involved in the export of polyamines, in particular of spermidine and spermine (Albertsen et al. 2003; Krüger et al. 2013; Uemura et al. 2005); however, this physiological role has, so far, not been linked to its role in MDR.

The extensive amount of information that has been gathered regarding the transcriptional regulatory networks underlying the control of the expression of yeast MFS-MDR encoding genes, largely compiled in the YEASTRACT database (Teixeira et al. 2006b, 2014), has been contributing to elucidate the function of these transporters in the MDR context and also outside of it, in a more physiological perspective. Key transcriptional regulators of MDR in yeast, such as Pdr1, Pdr3 and Yap1, were found to control drug-induced transcriptional activation of MFS-MDR-encoding genes (as recently reviewed in Dos Santos et al. 2014), including *TPO1* (Alenquer et al. 2006; do Valle Matta et al. 2001; Teixeira and Sá-Correia 2002). Nevertheless, a closer inspection of the data available in the YEASTRACT database shows that most yeast MFS-MDR-encoding genes have more documented regulatory associations with transcription factors not specifically linked to the MDR phenomenon than with those that are known to control MDR (Dos Santos et al. 2014). Gcn4, a transcription factor involved in signalling amino acid internal homeostasis, and Bas2, an activator of the histidine and purine biosynthetic pathways, stand out as they are associated to the transcriptional regulation of around 70% of all genes encoding MFS-MDR transporters in yeast (Dos Santos et al. 2014). In some cases, the transcriptional regulation by non-MDR transcription factors of MFS-MDR transporters is consistent with their proposed physiological function. This is the case of the Gcn4-regulated transporters Aqr1, implicated in the vesicle-mediated extrusion of homoserine, threonine and other amino acids (Velasco et al. 2004); Vba1–5, proposed to catalyse transport of amino acids into the vacuole (Shimazu et al. 2005), and Qdr2, which was also demonstrated to affect amino acid homeostasis (Vargas et al. 2007).

In this work, Tpo1 was identified as a determinant of yeast resistance to benzoic acid. A strong up-regulation of the *TPO1* gene was registered in response to benzoic acid stress; however, this was found to be independent of Pdr1 and other transcription factors specifically related with drug stress response and tolerance. Instead, the benzoic acid-induced transcriptional activation of *TPO1* was found to be dependent of Gcn4 and Stp1, which were also found to play an essential role in tolerance of *S. cerevisiae* to benzoic acid. Taking into consideration the crucial role played by the Gcn4- and Stp1-dependent pathways in yeast sensing and signalling of internal amino acid homeostasis (Ljungdahl and Daignan-Fornier 2012), the hypothesised involvement of Tpo1 in polyamines and in amino acid internal homeostasis under benzoic acid stress was dissected. The results obtained provide useful insights into the link between the intracellular homeostasis of nitrogenous compounds and Tpo1 regulation and protective role in response to benzoic acid.

## Materials and methods

### Strains and plasmids

The parental strain *S. cerevisiae* BY4741 (*MAT* ***a***, *ura3*Δ0, *leu2*Δ0, *his3*Δ1, *met15*Δ0) and the derived deletion mutants (BY4741_*tpo1*Δ, BY4741_*pdr1*Δ, BY4741_*pdr3*Δ, BY4741_*pdr8*Δ, BY4741_*yap1*Δ, BY4741_*yap2*Δ, BY4741_*yap3*Δ, BY4741_*yap4*Δ, BY4741_*yap5*Δ, BY4741_*war1*Δ, BY4741_*gcn4*Δ, BY4741_*stp1*Δ and BY4741_*stp2*Δ) were obtained from the Euroscarf collection. The amino acid prototrophic strain 23344c (*MAT* ***a***, *ura3*) was kindly provided by B. André (Université libre de Bruxelles, Belgium). *S. cerevisiae* BY4741 strains in which genome the vector pRS303*GPD* or the construction pRS303*GPD*_*TPO1* was integrated (herein referred to as BY4741.*GPD* and BY4741.*GPD*_*TPO1*, respectively) were kindly provided by M. Ralser (University of Cambridge, UK). The plasmids prepared and/or used in this study are listed in Table 1.

**Table 1:** List of plasmids used in this study

Plasmid name	Description	Reference
pYEP351	Yeast/*E. coli* shuttle vector with a LEU2 marker	Hill et al. (1986)
pYEP351_*TPO1*	Yeast/*E. coli* shuttle vector with a LEU2 marker, in which the *TPO1* gene was cloned	Tomitori et al. (2001)
p*TPO1*::*lacZ*	Expression fusion plasmid in which 1000 bp of the *TPO1* promoter region was fused with a *lacZ*-coding sequence at the pAJ152 basal vector	Alenquer et al. (2006)
pYEP354	Yeast episomal vector with URA3 marker for construction of lacZ fusions	Myers et al. (1986)
pYEP354_*TPO1*::*lacZ*	Expression fusion plasmid in which 1000 bp of the *TPO1* promoter region was fused with a *lacZ*-coding sequence at the pYEP354 basal vector	This study
p*YEP354_TPO1(GRE1* ^*^ *)*::*lacZ*	Plasmid derived from pYEP354_*TPO1*::*lacZ* in which the Gcn4-binding site TGACTC located at position −771 of the *TPO1* promoter region was replaced by TGAGGC	This study
p*YEP354_TPO1(GRE2* ^*^ *)*::*lacZ*	Plasmid derived from pYEP354_*TPO1*::*lacZ* in which the Gcn4-binding site TGACTC located at position −175 of the *TPO1* promoter region was replaced by TTTCTC	This study
p*YEP354_TPO1(SRE1* ^*^ *)*::*lacZ*	Plasmid derived from pYEP354_*TPO1*::*lacZ* in which the St1p-binding site CGGCTC located at position −630 of the *TPO1* promoter region was replaced by CGGATC	This study

### Growth media

Cells were batch-cultured at 30 °C, with orbital agitation (250 rpm), in MM4 growth media which contains, per litre, 20 g glucose (Merck, Darmstadt, Germany), 1.7 g yeast nitrogen base without amino acids or NH_4_^+^ (Difco, Detroit, Michigan, USA) and 2.65 g (NH_4_)_2_SO_4_ (Merck, Darmstadt, Germany). To cultivate BY4741 and the deletion mutant strains derived from BY4741, the MM4 growth medium was further supplemented with 20 mg/L methionine, 20 mg/L histidine, 60 mg/L leucine and 20 mg/L uracil (all from Sigma, Missouri, USA). The amino acid prototrophic strain 23344c was cultivated in MM4 growth medium supplemented with 20 mg/L uracil. Solid MM4 growth medium was obtained by supplementing the liquid medium with 2% agar (Iberagar, Barreiro, Portugal). The pH of liquid MM4 growth medium was adjusted to 4.0 using HCl as the acidulant. The stock solution of benzoic acid (potassium salt; Sigma, St. Louis, MO, USA) used to supplement the media was prepared in water, and the pH of this solution was adjusted to 4.0 using HCl.

### Benzoic acid susceptibility assays

The susceptibility of the *S. cerevisiae* strains tested to benzoic acid was examined by comparing the growth of these two strains in liquid medium. Mid-exponential cells (OD_600nm_ 0.5 ± 0.05) cultivated in liquid MM4 medium (at pH 4.0) were used to re-inoculate (at initial OD_600nm_ of 0.05) this same basal medium either or not supplemented with 0.9 mM benzoic acid. Growth in the presence or absence of benzoic acid was monitored by accompanying the increase in the OD_600nm_ of the cultures. Susceptibility of BY4741.*GPD* and BY4741.*GPD_TPO1* was assessed in a range of 0.7–1.1 mM benzoic acid, in the same minimal media. To assess the effect of pH established without the addition of any weak acid, the MM4 growth medium pH was adjusted in the range of 2–5 with HCl and NaOH.

### Subcultivation of benzoic acid-adapted yeast cells

*S cerevisiae* BY4741 and the derived deletion mutant *tpo1*Δ cells were cultivated until mid-exponential phase (OD_600nm_ = 0.6 ± 0.05) in liquid MM4 medium (at pH 4.0) and re-inoculated in this same basal medium supplemented with 0.9 mM benzoic acid. When cultures resumed growth, they were subcultivated in fresh MM4 medium supplemented with the same concentration of benzoic acid. Growth was followed by measuring OD_600nm_ and colony forming units.

### Measurement of *TPO1* expression based on *lacZ* fusions

*S. cerevisiae* BY4741 and the derived deletion mutants *pdr1*Δ, *pdr3*Δ, *pdr8*Δ, *yrr1*Δ, *yap1*Δ, *yap2*Δ, *yap3*Δ, *yap4*Δ, *yap5*Δ, *gcn4*Δ, *stp1*Δ, *stp2*Δ and *war1*Δ transformed with p*TPO1*::*lacZ* plasmid (Alenquer et al. 2006) were cultivated until mid-exponential phase (OD_600nm_ = 0.6 ± 0.01) in MM4 growth medium lacking uracil (at pH 4.0) and then re-inoculated (at an OD_600nm_ = 0.2 ± 0.01) into this same basal growth medium supplemented or not with 0.9 mM benzoic acid. The expression of the *TPO1* gene in the wild-type and in the mutant strains was compared in mid-exponential phase cells incubated for 12 h in the presence of benzoic acid. This time-point was found to lead to maximum expression of the *TPO1* gene measured from the p*TPO1*::*lacZ* plasmid in wild-type benzoic acid-challenged cells. In control cultures, cells were harvested in the mid-exponential phase of growth (6 h of incubation in MM4 growth medium). The determination of β-galactosidase activity was carried out as described before (Alenquer et al. 2006), the enzyme specific activity units (U; Miller units) being defined as the increase in A_420_ per minute (OD_600nm_)^−1^ × 1000.

To examine the effect that the Gcn4 response element (GRE) or the Stp1 response element (SRE) motifs present in the *TPO1* promoter have in the expression of the *TPO1* gene under benzoic acid stress, these DNA motifs were inactivated by site-directed mutagenesis using as a template pYEP354w_*TPO1*::*lacZ* fusion plasmid. This plasmid was constructed by cloning the promoter region of *TPO1* (considered as the 1000 bp ustream of the start codon) into the *Bam*HI and *Pst*I sites of the pYEP354w vector. The p*TPO1*::*lacZ* plasmid could not be used as template in the mutagenic PCR reactions due to its high molecular weight (∼14 kb), and thus, a shorter *lacZ* fusion, based on the YEP354w vector (∼6 kb), was constructed for this purpose. *lacZ* expression from pYEP354_*TPO1*::*lacZ*, pYEP354_*TPO1*(GRE1)::*lacZ*, pYEP354_*TPO1*(GRE2)::*lacZ* and pYEP354_*TPO1*(SRE2)*::lacZ* plasmids was assessed by real-time RT-PCR. For that, cell samples were obtained by centrifugation [5000 rpm in a Beckman (Brea, California, USA) JA20 rotor, 4 °C, 5 min] and immediately frozen at −80 °C until total RNA extraction. One microgram of total RNA was used for complementary DNA (cDNA) synthesis. The reverse transcription step was performed using the multiscribe reverse transcriptase kit (Applied Biosystems, Foster City, California, USA) in a 7500 RT-PCR thermal cycler block (Applied Biosystems, Foster City, California, USA). Approximately 10 ng of the synthesized cDNA was used for the subsequent PCR step. In all experiments, the transcript level of *ACT1* messenger RNA (mRNA) was used as an internal control. The primers used for amplification of *ACT1* cDNA (3′-CTCCACCACTGCTGAAAGAGAA-5′, 5′- CCAAGGCGACGTAACATAGTTTT-3′) and of *lacZ* cDNA (3′-AAAGCTGCAAGTCTGCATCACAC-5′ and 5′-GCACGATAGAGATTCGGGATTT-3′) were designed using Primer Express Software (Applied Biosystems, Foster City, California, USA). The relative values obtained for the expression from the native promoter in control conditions were set as 1, and the remaining values presented are relative to that control.

### Measurement of *TPO1* transcription based on real-time RT-PCR

Real-time RT-PCR was used to compare *TPO1* mRNA levels during cultivation of *S. cerevisiae* BY4741 cells or of the deletion mutants *pdr1*Δ, *pdr3*Δ, *stp1*Δ, *stp2*Δ and *gcn4*Δ in MM4 growth medium (at pH 4.0) either or not supplemented with benzoic acid (0.9 mM), as described in the previous section. The primers used for the amplification of the probes selected to monitor *TPO1* (3′-TCTGACAATTCACTACCGAACAATC-5′, 5′-GGCGTGCCGCTGCTT-3′) and *ACT1* expression (the same as indicated in the previous section) were designed using Primer Express Software (Applied Biosystems, Foster City, California, USA). The specificity of the probe selected for monitoring *TPO1* transcription was confirmed by the absence of an amplification product in the *tpo1*Δ mutant. The relative values obtained for the wild-type strain in control conditions were set as 1, and the remaining values presented are relative to that control. A similar experimental setup was used to monitor *TPO1* transcription in conditions of leucine exhaustion during growth of wild-type and *gcn4*Δ cells in MM4 growth medium or during growth of the 2344c strain in MM4 growth medium having a limiting (0.00265 g/L) or a saturating (2.65 g/L) concentration of ammonium.

### Quantification of intracellular concentration of polyamines and amino acids

The intracellular concentration of amino acids and polyamines in wild-type or Δ*tpo1* cells was compared after 1 h of incubation in MM4 growth medium (at pH 4.0) either or not supplemented with 0.9 mM benzoic acid. Yeast cells were harvested by centrifugation (5000 rpm in a Beckman JA20 rotor, 4 °C, 5 min), washed two times with ice-cold distilled water and frozen at −80 °C until further use. Polyamine extraction was performed by re-suspending the harvested cells in 600 μL 10% trichloroacetic acid (TCA) supplemented with 1 mM 1,6-diaminohexane (Fluka, Buchs, Switzerland) which was used as the internal standard. The cell suspension obtained was incubated at 70 °C for 1 h, centrifuged for 5 min (at 13000 *g*), and the supernatant was recovered into a new tube. Quantification of the content of spermine, spermidine and putrescine present in the 600-μL supernatant recovered was determined, by HPLC, as a service at the Instituto Biologia Experimental e Tecnológica (Oeiras, Portugal). The method used had a detection limit of 5 μM.

Intracellular amino acid pools were obtained using the method described before (Klasson et al. 1999). Briefly, yeast cells were harvested in the same conditions as those used for polyamine quantification and were washed twice with 1.5 mL of water and re-suspended in 1.5 mL of AA buffer (2.5 mM K_2_HPO_4_-KH_2_PO_4_ at pH 6.0; 0.6 M sorbitol; 10 mM glucose). The washed filters were boiled in 3 mL of water for 15 min. One-millilitre aliquots of this suspension were taken and centrifuged to remove particles of filter. The concentrations of amino acids present in this 1 mL sample were determined, by HPLC, as a service at the Laboratório Nacional Dr. Ricardo Jorge (Lisbon, Portugal). The method used had a detection limit ranging between 0.1 and 0.5 μg/mL for the different amino acids.

## Results

### TPO1 *is a determinant of yeast resistance to benzoic acid*

**Fig. 1:**
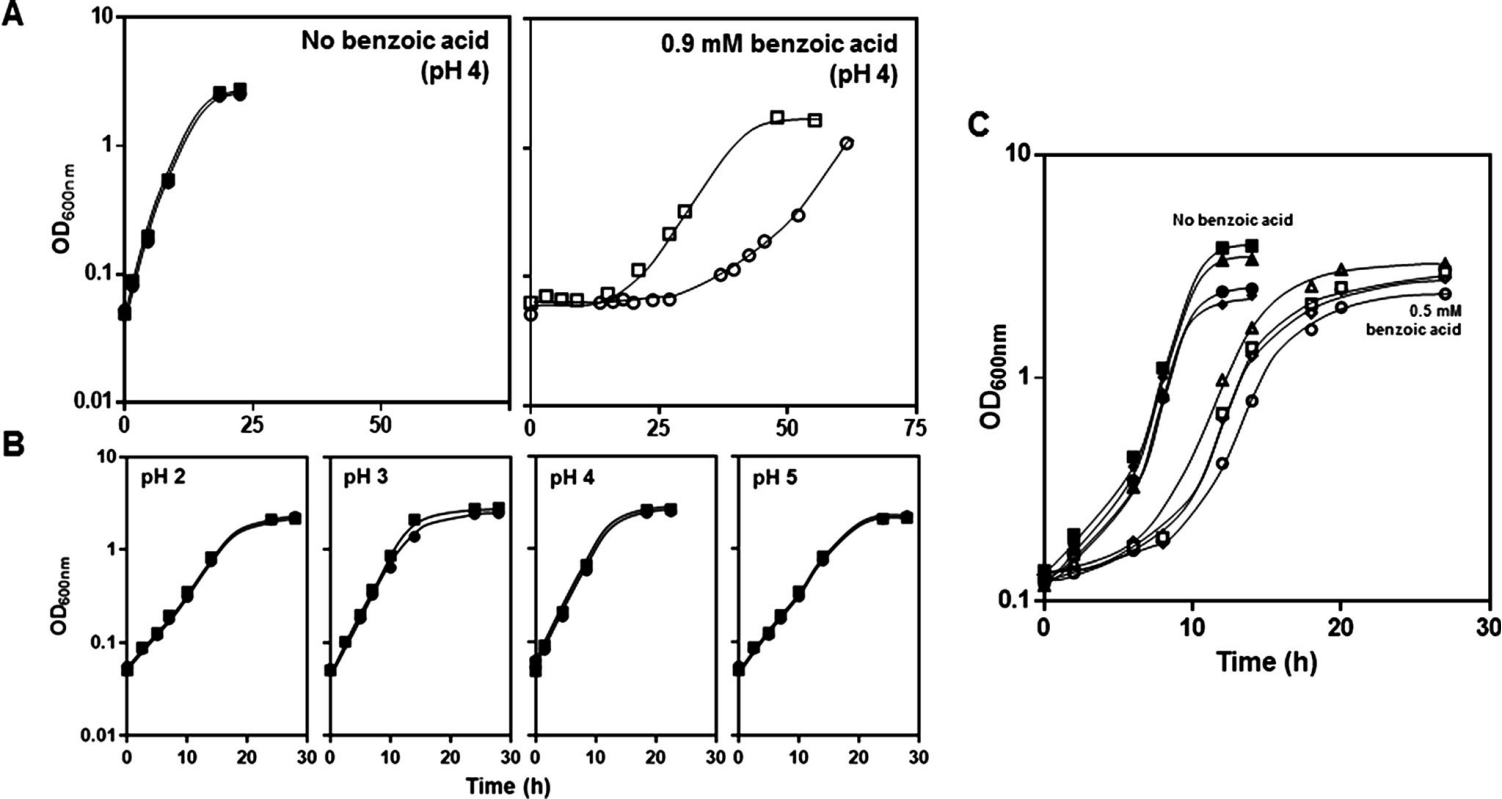
*TPO1* expression is required for a more rapid yeast adaptation to benzoic acid stress but not to low pH when a strong acid is used as the acidulant. **a** Growth curves of *S. cerevisiae* BY4741 (*filled square*, *open square*) or of the derived deletion mutant *tpo1*Δ (*filled circle*, *open circle*) in MM4 growth medium (at pH 4.0) either (*open symbols*) or not (*closed symbols*) supplemented with benzoic acid (0.9 mM) or **b** in this same basal medium acidified at pH 2, 3.5 or 5 using HCl as the acidulant (*lower panel*). **c** Growth curves of *S. cerevisiae* BY4741 and *tpo1*Δ harbouring an empty vector (*open square* and *open circle*, respectively) or the same pYEP351 vector with the *TPO1* gene cloned (*open triangle* and *open diamond*, respectively) supplemented (*open symbols*) or not (*closed symbols*) with 0.5 mM benzoic acid. The growth curves shown are representative of, at least, three independent experiments that gave rise to the same growth patterns

The comparison of the growth curves of unadapted cell populations of the parental strain *S. cerevisiae* BY4741 and of the *tpo1*Δ derived mutant in MM4 medium supplemented with 0.9 mM benzoic acid (at pH 4.0) shows that the elimination of *TPO1* significantly increases the duration of the adaptation period to the acid (from 18 h to approximately 30 h) and reduces the maximum specific growth rate of the adapted cell population (from 0.097 to 0.057 h^−1^) (Fig. 1a). However, *TPO1* deletion had no detectable effect in yeast growth during cultivation in MM4 growth medium acidified to pH 2.0 using a strong acid (HCl) as the acidulant (Fig. 1b). This result shows that Tpo1 is specifically required for protection against benzoic acid and not against low pH by itself.

Expression of *TPO1* from a centromeric plasmid (pYEP351_*TPO1*) was further found to rescue the benzoic acid susceptibility exhibited by the *tpo1*Δ deletion mutant, enabling this mutant strain to display a susceptibility profile similar to the one exhibited by the BY4741 wild-type strain harbouring the empty vector pYEP351 (Fig. 1c). Insertion of pYEP351_*TPO1* in the wild-type strain resulted in improved resistance to exposure to benzoic acid (Fig. 1c).

**Fig. 2:**
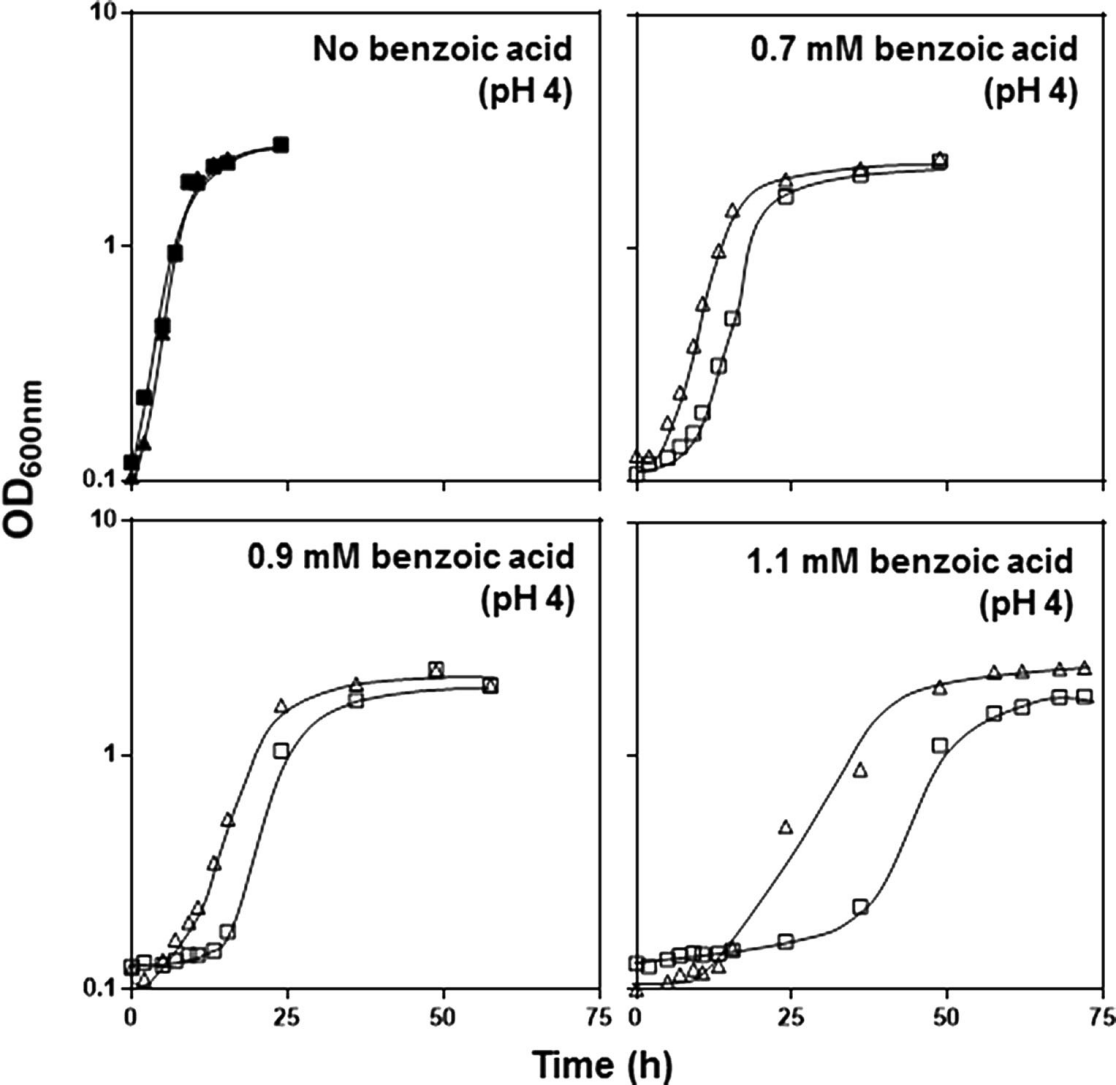
*TPO1* overexpression leads to an enhanced tolerance to benzoic acid induced stress. Growth curves of *S. cerevisiae* BY4741.*GPD* (*filled square*, *open square*) and BY4741.*GPD*_*TPO1* (*filled triangle*, *open triangle*) in MM4 growth medium (pH 4.0) either or not supplemented with benzoic acid (0.7–1.1 mM). The growth curves shown are representative of, at least, three independent experiments that gave rise to the same results

The BY4741.*GPD*_*TPO1* strain (Krüger et al. 2013), overexpressing the *TPO1* gene by insertion of an extra copy in the genome controlled by the strongest constitutive yeast promoter *GPD*, proved to be less susceptible to benzoic acid stress in all the concentrations tested when compared to BY4741.*GPD*, the respective wild-type parental strain (Fig. 2). In fact, overexpression of *TPO1* proved to reduce the adaptation phase of yeast cells to half when exposed to a very high benzoic acid concentration (1.1 mM), also rendering a higher final biomass when compared to the BY4741.*GPD* cell culture (Fig. 2).

**Fig. 3:**
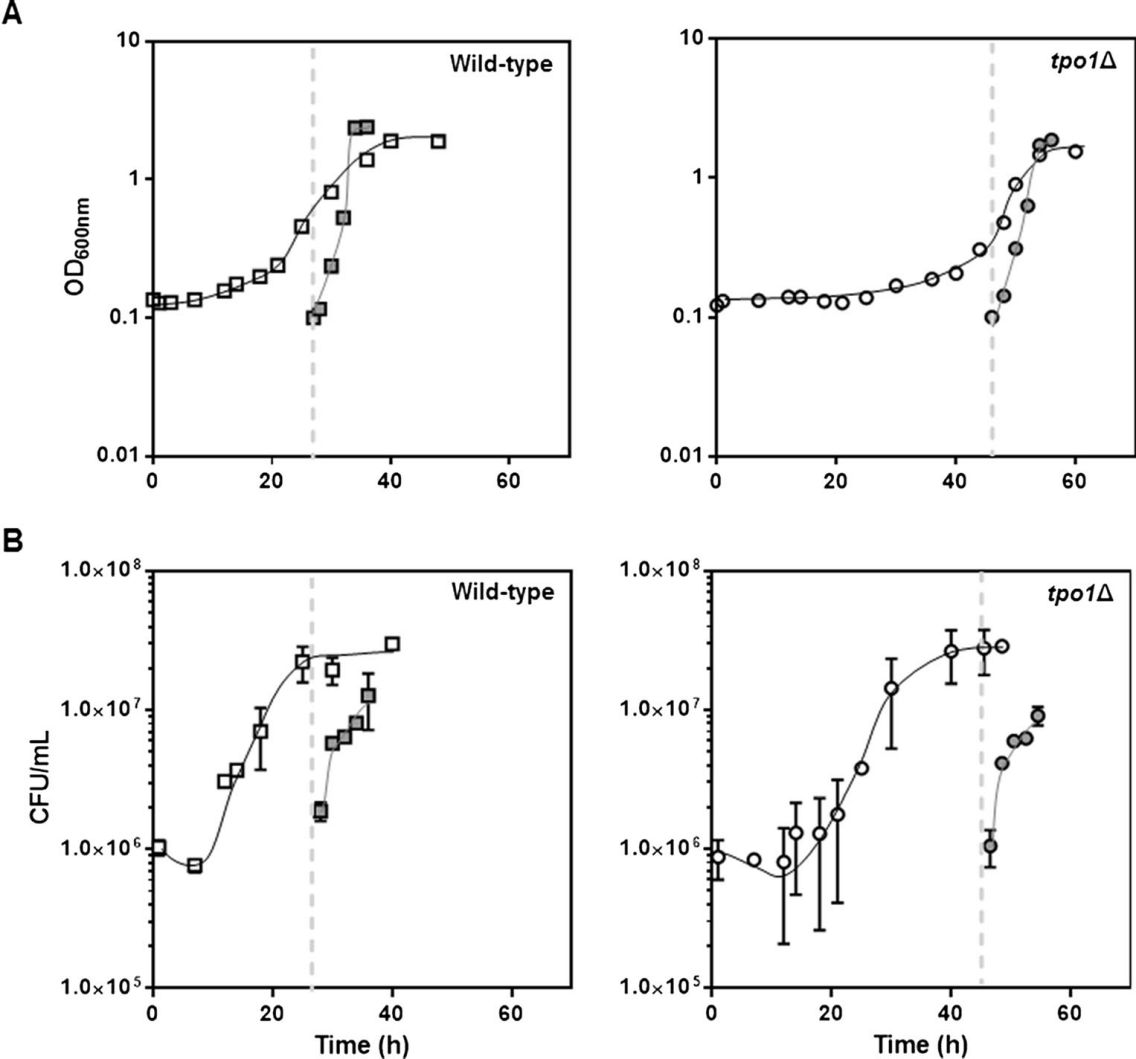
Subcultivation of benzoic acid-adapted cells shows yeast cell adaptation independently of Tpo1 expression. Growth curves of wild-type (*open square*, *grey square*) and derived deletion mutant *tpo1*Δ (*open circle*, *grey circle*) in the presence of 0.9 mM benzoic acid (*open symbols*) in MM4 (pH 4.0). *Grey symbols* represent the subcultivation in fresh medium MM4 supplemented with 0.9 mM benzoic acid of cells harvested in the time-point marked with the *dashed line*. Cells cultures were followed by measuring OD_600nm_ (**a**) and colony forming units per millilitre (**b**). The results are representative of, at least, three independent experiments, and error bars represent standard deviation

The ability of wild-type and *tpo1*Δ mutant cells to adapt to benzoic acid-induced stress was tested by harvesting yeast cultures that resumed growth after a latency phase in MM4 media supplemented with 0.9 mM benzoic acid and re-inoculating them in fresh media with the same benzoic acid concentration. Pre-adapted cell populations did not exhibit a lag-phase period when exposed for the second time to the same stress (Fig. 3a). Although benzoic acid stress seems to induce a more severe and longer period of loss of viable cells in *tpo1*Δ cultures, the subcultivation of the adapted population rendered a similar growth in both wild-type and *tpo1*Δ cell cultures (Fig. 3b), consistent with that observed in optical density measurements. This phenomenon suggests that the role of Tpo1 is predominantly sensed in the period of adaptation to sudden benzoic acid, enabling the population to adapt faster to the presence of this food preservative.

### TPO1 *transcription is activated under benzoic acid stress in the dependence of Gcn4 and Stp1 transcription factors*

**Fig. 4:**
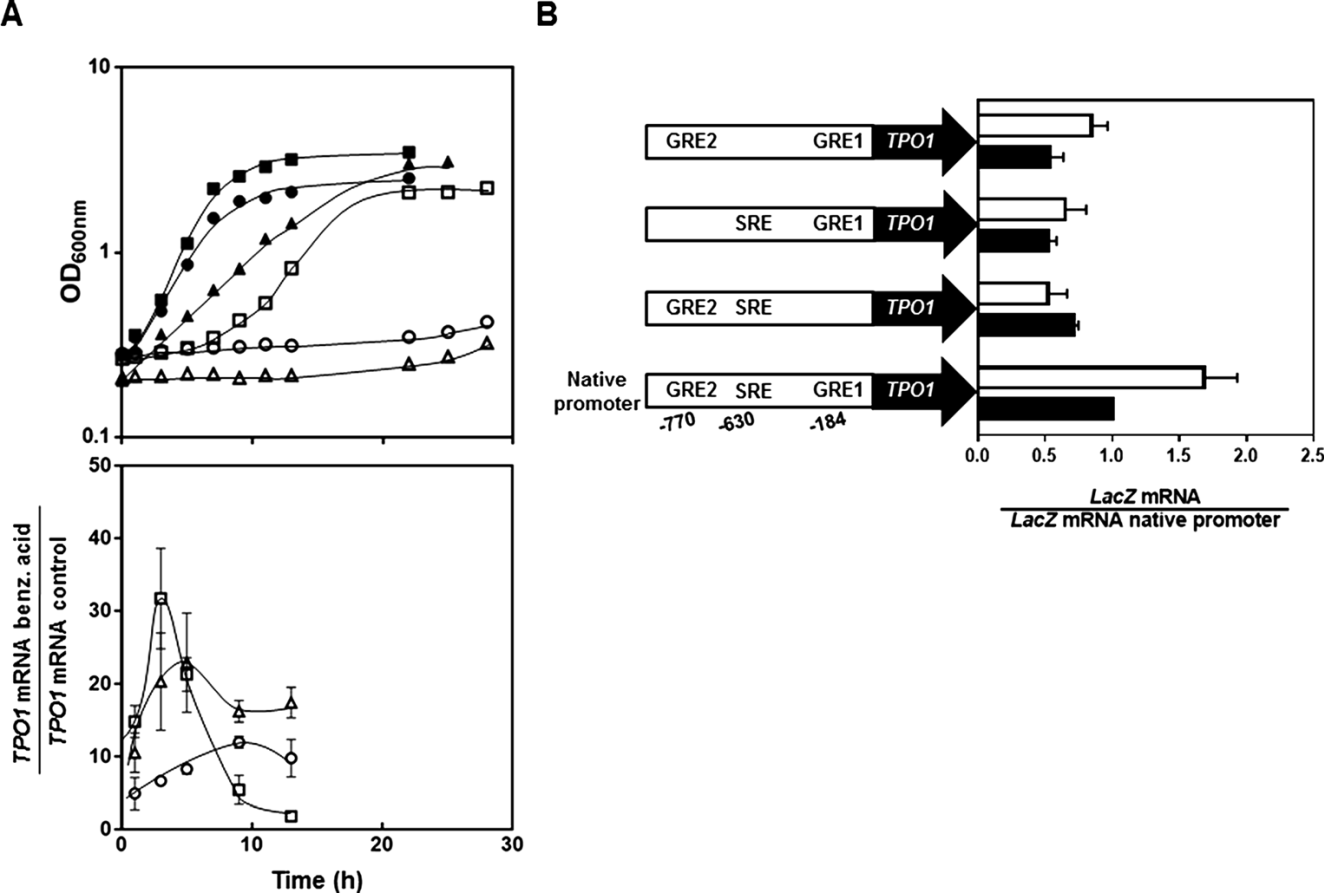
*TPO1* is up-regulated under benzoic acid stress in a Gcn4- and Stp1-dependent manner **a** Growth curve of *S. cerevisiae* BY4741 (*filled square*, *open square*) and of the deletion mutants BY4741_*gcn4*Δ (*filled triangle*, *open triangle*) and BY4741_*stp1*Δ (*filled circle*, *open circle*) in MM4 growth medium (at pH 4.0) (*closed symbols*) or in this same basal medium supplemented with 0.9 mM benzoic acid (*open symbols*). Quantification of *TPO1* mRNA levels during the growth curve of the three strains in the presence or absence of benzoic acid was based on quantitative real-time RT-PCR. For each strain, the transcript levels of the *TPO1* gene shown are relative to the transcript levels registered in exponential-phase cells (at an OD_600nm_ of 0.4) cultivated in unsupplemented MM4 growth medium (at pH 4.0). In all samples, *TPO1* mRNA levels were normalized using *ACT1* transcript levels. **b** Effect of the Gcn4 response elements (GRE) and Stp1 response elements (SRE) located in the *TPO1* promoter in the benzoic acid-induced up-regulation of *TPO1*, measured, through RT-PCR, as the mRNA level of the *LacZ* gene, expressed under the control of the *TPO1* promoter. Wild-type cells harbouring the pYEP354w_*TPO1*::*lacZ* plasmid (which contains natural *TPO1* promoter) or the derived mutant constructs having the GRE and SRE motifs individually inactivated were cultivated in MM4 growth medium (at pH 4) (*black bars*) or in this same growth medium supplemented with 0.9 mM benzoic acid (*white bars*) and harvested after 3 h of growth

A dramatic increase (up to 30-fold) in *TPO1* transcript levels was registered during cultivation of an unadapted *S. cerevisiae* BY4741 population in the presence of a growth-inhibiting concentration of benzoic acid (0.9 mM at pH 4.0) compared with cells grown in unsupplemented medium (control cells) (Fig. 4a). This strong, but transient, stimulation of *TPO1* transcript levels reached a maximum value during the period of latency induced by benzoic acid, after 3 h of incubation to the acid, after which mRNA levels decreased steeply to basal levels once adapted cells resumed exponential growth (Fig. 4a).

The *TPO1* promoter region harbours at least each one binding site for 12 transcription factors known to be involved in yeast response to stress and other environmental challenges: Pdr1, Pdr3, Pdr8, Yrr1, Yap1, Yap2, Yap3, Yap4, Yap5, Msn2, Msn4, War1, Gcn4, Stp1 and Stp2 (Fig. S1 in the Supplementary Material). To examine the role of these transcription factors in benzoic acid-induced up-regulation of *TPO1* expression, the parental strain and several deletion mutants individually lacking these regulators were transformed with the p*TPO1*::*lacZ* fusion plasmid (Alenquer et al. 2006). The levels of β-galactosidase produced in benzoic acid-stressed cells harbouring the p*TPO1*::*lacZ* plasmid were only found to be reduced, but not fully abrogated, in mutants devoid of Gcn4 or Stp1 transcription factors (Fig. S1 in the Supplementary Material). Consistent with this observation, *TPO1* mRNA levels produced in benzoic acid-challenged *gcn4*Δ and *stp1*Δ populations were significantly below the levels registered in the parental strain (Fig. 4a). Indeed, *TPO1* transcription in the *gcn4*Δ and *stp1*Δ mutant strains was found to reach only a 20-fold transient activation under benzoic acid stress, which represents a 30% reduction in the maximum level of *TPO1* up-regulation registered in the wild-type strain. Also, the period of time during *TPO1* up-regulation appears to be extended in the mutant strains, possibly due to the fact that the lag phase induced by benzoic acid in the mutant cell populations is also much longer than the one registered for the wild-type strain. The undetectable effect of Pdr1, Pdr3 and Stp2 in the activation of *TPO1* transcription under benzoic acid stress was also confirmed by real-time RT-PCR (results not shown). To know if the effect of Gcn4 and Stp1 on *TPO1* transcription is direct, the three predicted Gcn4/Stp1 DNA-binding sites found in the *TPO1* promoter (two for Gcn4 and one for Stp1, designated GRE and SRE motifs) were individually removed in the pYEP354w:*TPO1 lacZ* fusion by mutagenesis, after which the responsiveness of the mutagenized constructs to benzoic acid stress was assessed by measurement of *lacZ* mRNA by RT-PCR (Fig. 4b). Inactivation of either of the two Gcn4-binding sites reduced the benzoic acid-induced up-regulation of the *TPO1* gene (Fig. 4b). Inactivation of the Stp1-binding site also abrogated the benzoic acid-induced up-regulation of *TPO1* (Fig. 4b). Significantly, even in the absence of benzoic acid, the inactivation of the Gcn4- and Stp1-binding sites had a moderate effect in the *TPO1* transcription level (Fig. 4b). Altogether, the results obtained are consistent with the concept that under benzoic acid stress, *TPO1* overexpression is under the coordinated action of Gcn4 and Stp1. Also, elimination of Gcn4 or Stp1 led to a dramatic increase in yeast susceptibility to benzoic acid, even higher than the one obtained upon deletion of *TPO1* (Fig. 4a), suggesting that there are other determinants of yeast tolerance to benzoic acid among the Gcn4 and Stp1 target genes.

### Effect of Tpo1 expression and benzoic acid stress in internal homeostasis of polyamines and amino acids

**Fig. 5:**
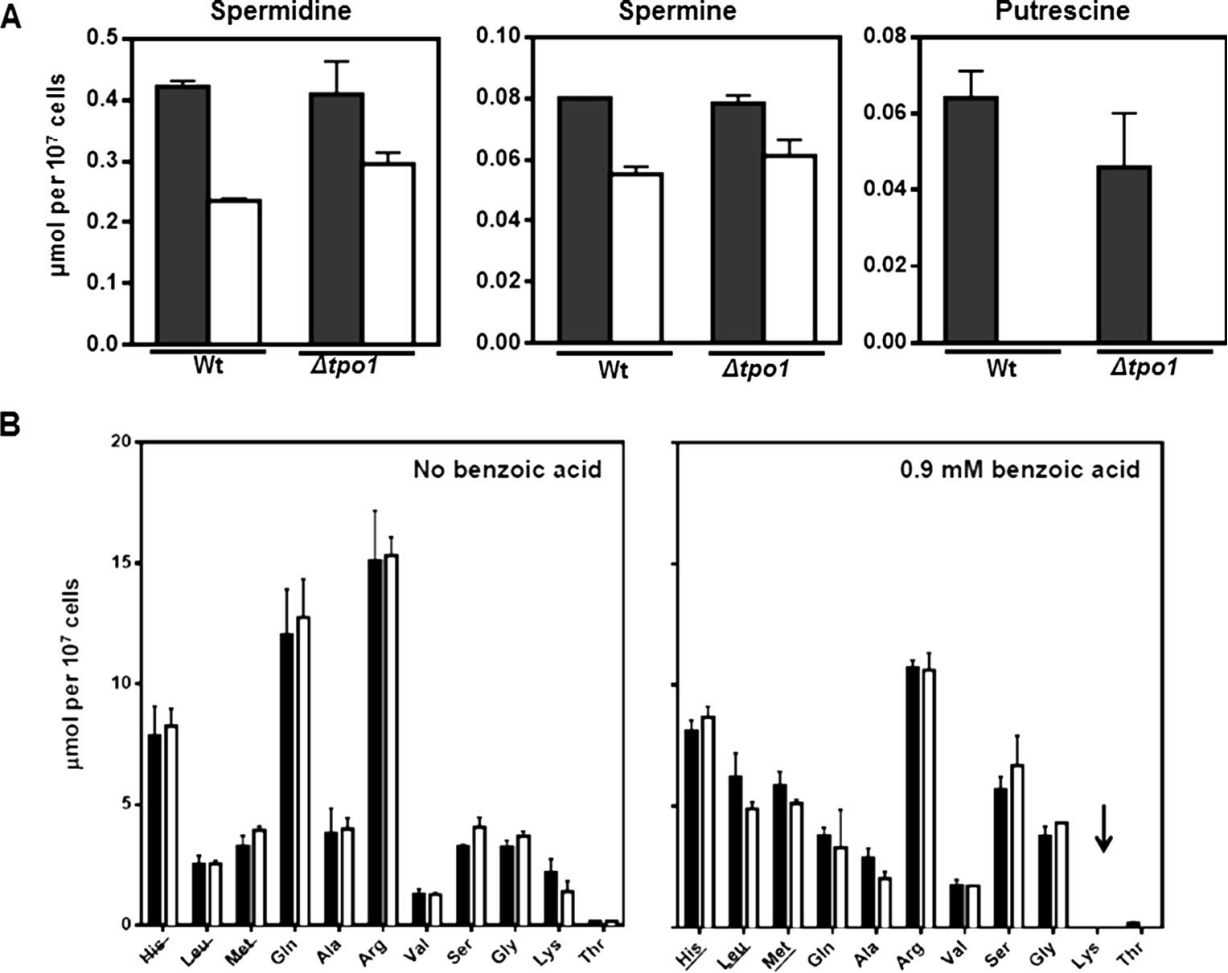
Effect of benzoic acid stress and *TPO1* expression in the internal pool of polyamines and amino acids**. a** Spermidine, spermine and putrescine intracellular levels after 1 h of incubation in the absence (*black bars*) or presence (*white bars*) of benzoic acid (0.9 mM). **b** Intracellular amino acid pools of BY4741 (*black bars*) and BY4741_*tpo1*Δ (*white bars*) in the presence or absence of 0.9 mM benzoic acid. The amino acids added to the MM4 growth medium to suppress the auxotrophies of the BY4741 strain are *underlined*. The *arrows* indicate amino acids whose concentration was below the detection limit in the acid-challenged cells. *Wt* wild-type

The effect of *TPO1* expression and of benzoic acid stress in the internal concentration of polyamines was also examined considering the described involvement of Tpo1 in the export of these nitrogenous compounds (Albertsen et al. 2003; Krüger et al. 2013; Tomitori et al. 2001) (Fig. 5). Since benzoic acid-induced up-regulation of *TPO1* transcription was found to be controlled by Gcn4 and Stp1 and these transcription factors are key regulators of amino acid sensing and signalling in yeast (Ljungdahl and Daignan-Fornier 2012), we also examined the effect of Tpo1 expression in internal amino acid homeostasis, either in the presence or in the absence of benzoic acid stress (Fig. 5). For this, the internal pools of amino acids and polyamines recovered from wild-type and *tpo1*Δ cells cultivated for 1 h in the presence or absence of benzoic acid (0.9 mM, at pH 4) were compared by HPLC, these being the same experimental conditions that were found to lead to the strong transcriptional activation of *TPO1* mediated by Gcn4 and Stp1 (Fig. 5).

The internal concentration of putrescine, spermine and spermidine was found to be similar in wild-type and *tpo1*Δ cells in the exponential phase of growth in MM4 growth medium (Fig. 5a). Exposure, for 1 h, of unadapted BY4741 cell population to benzoic acid stress (0.9 mM, at pH 4.0), corresponding to the early period of adaptation to the acid, led to a reduction in the internal pool of the three polyamines, when compared to the values registered for control cell cultures (Fig. 5a). In fact, the putrescine levels fell under the detection limit. Surprisingly, no significant differences were observed between the wild-type and *tpo1*Δ yeast cell cultures regarding any of the polyamine intracellular levels determined (Fig. 5a).

The parental and *tpo1*Δ strains were found to exhibit, in general, similar amino acid pools (Fig. 5b). After 1 h of exposure to 0.9 mM benzoic acid (at pH 4.0), the intracellular pool of several amino acids decreased in the parental strain and in the *tpo1*Δ mutant, in particular glutamine, arginine and lysine, the internal concentration of lysine having decreased to levels below the detection limit (Fig. 5b). The exception to this pattern of reduction of intracellular amino acid concentration was the amino acids corresponding to the yeast strain auxotrophies (histidine, leucine and methionine) and that, for this reason, were supplemented to the growth medium (Fig. 5b). The intracellular levels of asparagine, isoleucine, proline, cysteine, phenylalanine and tyrosine could not be determined because they were below the detection limit of the technique. This could be the result of the cultivation of yeast cells in minimal growth medium (Kitamoto et al. 1988).

### TPO1 transcription is up-regulated in response to amino acid and nitrogen limitation

**Fig. 6:**
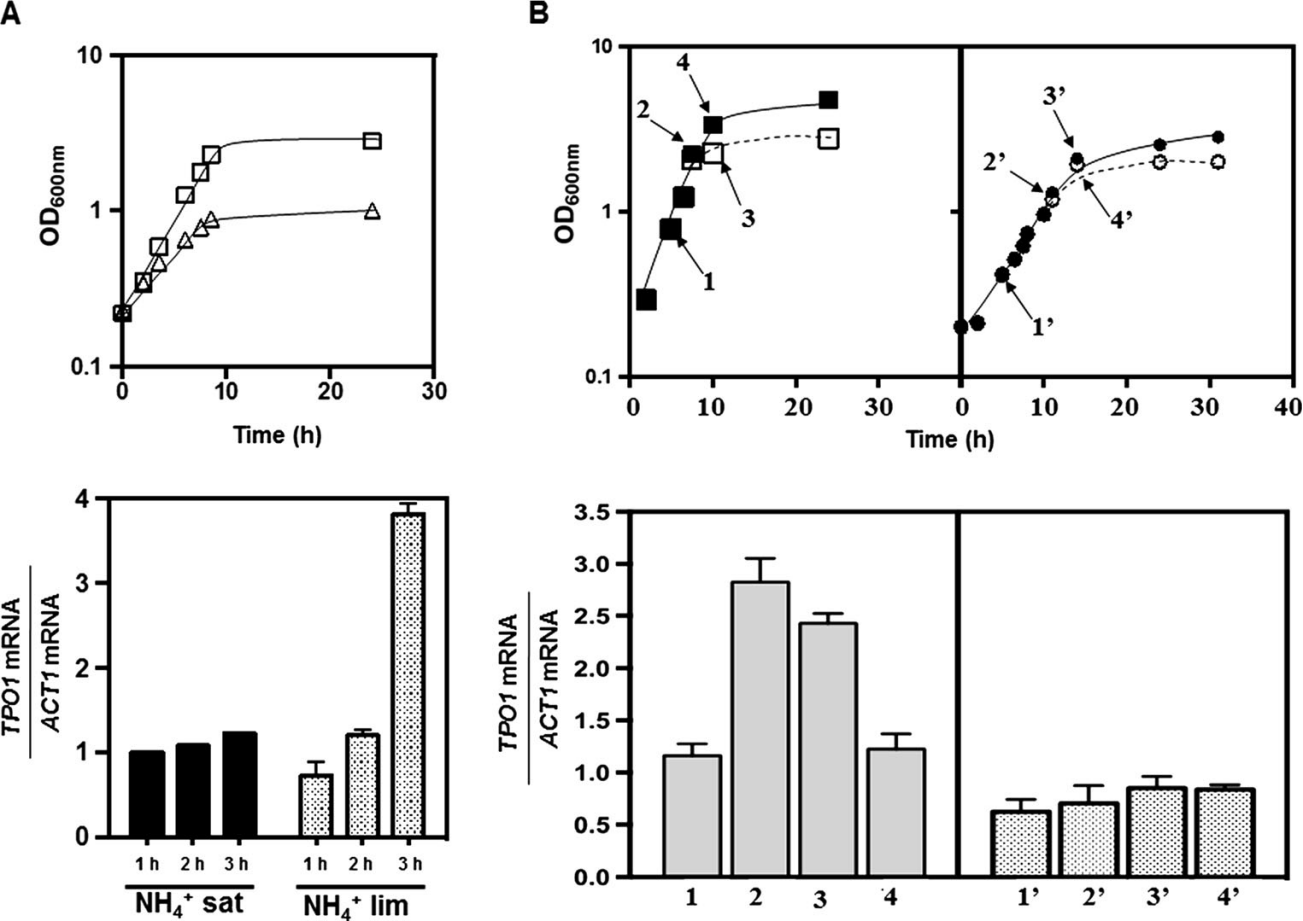
*TPO1* is up-regulated under ammonium or leucine limitation. **a** Expression of the *TPO1* gene during the first 3 h of growth of the prototrophic strain 2344c in minimal growth medium supplemented with a limiting (lim; *open triangle* and *grey bars*) (0.0265 g L^−1^) or a saturating (sat; *open square* and *black bars*) (2.65 g L^−1^) concentration of ammonium sulphate as the sole nitrogen source. Growth curve of the 2344c strain in the two conditions of ammonium availability is also shown. **b** Expression of *TPO1* during growth of *S. cerevisiae* BY4741 (*filled square*, *open square*) or of *gcn4*Δ (*filled circle*, *open circle*) in MM4 growth medium. Cell samples were harvested during exponential growth (sample 1) and when cells approached the stationary phase at an OD_600nm_ of 1.0 (sample 2). At this point, the cultures were split in two, and fresh leucine was added to one of the cultures (*closed symbols*) while the other remained in the leucine-exhausted growth medium (*open symbols*, *dashed curve*). After 1 h of incubation in the leucine-exhausted (sample 3) or in the leucine-replete growth medium (sample 4), cells were harvested and *TPO1* transcription levels were assessed. The results presented are means of at least three independent experiments, and error bars represent standard deviation

The transcription level of yeast MFS-MDR transporter-encoding genes *QDR2*, *AQR1* and *QDR3*, also required for resistance to polyamines, has been found to increase in response to amino acid and nitrogen limitation in a Gcn4-dependent manner (Dos Santos et al. 2014; Sá-Correia et al. 2009; Teixeira et al. 2011a). This indication prompted us to examine whether *TPO1* transcription was also responsive to this type of physiological perturbation (Fig. 6). The results obtained show that cells of the prototrophic strain 2344c cultivated for 3 h in minimal medium, supplemented with a limiting concentration of ammonium sulphate as the sole nitrogen source (0.0265 g L^−1^), exhibit a 3-fold higher expression of *TPO1*, compared with cells cultivated under the same conditions in a saturating concentration of ammonium (2.65 g L^−1^) (Fig. 6). *TPO1* transcript levels were also found to increase (by around 3-fold) when *S. cerevisiae* BY4741 cells entered the stationary phase of growth in the MM4 medium due to leucine exhaustion (Fig. 6). Leucine is one of the amino acids that have to be added to the growth medium to complement the auxotrophies of the BY4741 strain, and the concentration used in the MM4 medium, 60 mg L^−1^, was demonstrated to be growth-limiting (Vargas et al. 2007). Indeed, supplementation of the exhausted growth medium with fresh leucine restored growth of BY4741 cells and resulted in a decrease in the level of *TPO1* mRNA to values similar to those registered in the leucine-replete growth medium (Fig. 6). No significant increase in *TPO1* expression induced by leucine exhaustion during growth in the MM4 growth medium was observed in the *gcn4*Δ mutant (Fig. 6).

## Discussion

In this work, the *S. cerevisiae* plasma membrane drug:H^+^ antiporter Tpo1 was implicated for the first time in yeast tolerance to benzoic acid. Tpo1 was shown to be predominantly required in the period of adaptation to sudden benzoic acid, enabling the population to adapt faster to the presence of this food preservative. These results are actually consistent with previous observations, showing that Tpo1 (Teixeira and Sá-Correia 2002; Alenquer et al. 2006) and many other drug transporters are key players in the early response to sudden stress (Sá-Correia et al. 2009). Upon exposure to weak acids, such as acetic acid or 2-4-dichlorophenoxyacetic acid, it was demonstrated that together with the transient overexpression of drug transporters during the stress-induced lag phase, cells appear to activate additional mechanisms involved in cell wall and plasma membrane remodelling that decrease the permeability of the cell envelope (Teixeira et al. 2006a, 2006b, 2007; Simões et al. 2003; Viegas et al. 2005; Mira et al. 2010b, 2010c), avoiding a futile and energetically expensive cycle, in which the acid diffuses back into the cells, counteracting the active expulsion of its counterion.

**Fig. 7:**
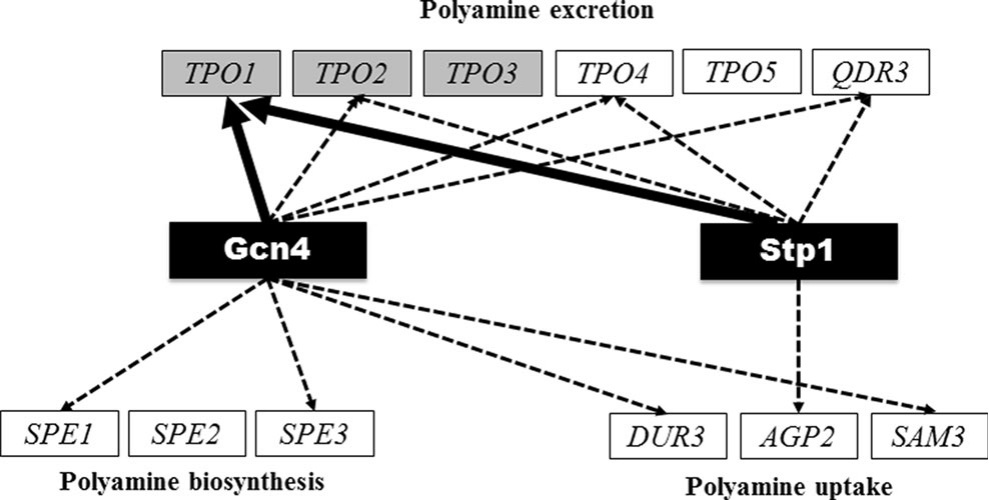
Effect of Gcn4 and Stp1 in the regulation of genes involved in synthesis, uptake and excretion of polyamines. Documented regulatory associations between the genes involved in synthesis, uptake and excretion of polyamines and Gcn4 and Stp1 are shown according with the information available in the YEASTRACT database (*dashed lines*), plus the data described herein on the effect of Stp1 and Gcn4 over *TPO1* expression (*full lines*). Genes up-regulated by benzoic acid stress are highlighted in *grey boxes* while down-regulated genes are shown in *white boxes* (Abbott et al. 2007)

During the adaptive response to benzoic acid stress, *TPO1* transcription was found to be strongly up-regulated (up to 30-fold), this activation being partially dependent on the Gcn4 and Stp1 transcription factors. Elimination of Gcn4 and/or Stp1 led to a dramatic increase in yeast susceptibility to benzoic acid, even higher than the one obtained upon deletion of *TPO1*, suggesting that there are other determinants of yeast tolerance to benzoic acid among Gcn4 and Stp1 target genes. The demonstration that none of the stress-responsive transcription factors that have a binding site in the *TPO1* promoter (Pdr1, Pdr3, Yrr1, Msn2, Msn4, Yap1, Yap2, Yap3, Yap4 and Yap5) are involved in the regulation of *TPO1* transcriptional activation under benzoic acid stress was surprising. In particular, it was unexpected to observe the lack of effect of Pdr1 in this activation, since this transcription factor is known to mediate all the previously described drug/xenobiotic-induced up-regulations of *TPO1* (Alenquer et al. 2006; do Valle Matta et al. 2001; Lucau-Danila et al. 2005; Teixeira and Sá-Correia 2002). Since Pdr1 function appears to be activated upon direct binding of xenobiotics/drugs (Thakur et al. 2008), it is possible that this lack of Pdr1 in the control of the benzoic acid response might result from the inability of benzoic acid (or benzoate) to bind to this protein. Interestingly, while the transcriptional association between Stp1 and *TPO1* is described here for the first time, Gcn4 had already been seen to play a role in the up-regulation of *TPO1*, in cells exposed to stress induced by 3-aminotriazole, a drug that mimics the effect of histidine limitation (Moxley et al. 2009). It is also interesting to observe that both Gcn4 and Stp1 have been documented to regulate the expression of several other genes involved in polyamine biosynthesis and transport (Fig. 7). The regulatory network schematized in Fig. 7 rejoins the information available in the YEASTRACT database (Teixeira et al. 2006a, 2006b, 2014) with the new data obtained in this study, and shows that Stp1 and/or Gcn4 are involved in the regulation of both uptake, biosynthesis and excretion of polyamines. Indeed, they are involved in the regulation of *TPO1* [this work and Moxley et al. (2009)] and, according to the data gathered in the YEASTRACT database, of its orthologues *TPO2*, *TPO3* and *TPO4*, required for polyamine excretion (Albertsen et al. 2003; Tomitori et al. 2001; Uemura et al. 2005), as well as *AGP2*, *DUR3* and *SAM1*, which mediate polyamine uptake (Aouida et al. 2013; Uemura et al. 2007). Despite the fact that benzoic acid-induced transcriptional activation of *TPO2* and *TPO3* is fully dependent of Haa1 (Fernandes et al. 2005), this transcription factor was found to have no effect in the regulation of *TPO1* transcription under the same conditions (our unpublished results).

The regulatory association between *TPO1* and Stp1 and Gcn4 is consistent with the idea that the transcriptional control of *TPO1*, as well as other MFS-MDR transporter-encoding genes, under stress may involve more than just transcription factors directly related to the response to drugs/xenobiotics/stress conditions (Dos Santos et al. 2014). This notion is further reinforced by previous observations showing that the regulators of methionine and leucine biosynthesis, Leu3 and Met32, and the activator of oleate catabolism, Pip2, are other non-MDR transcription factors described as regulators of *TPO1* expression (Carrillo et al. 2012; Smith et al. 2007; Tang et al. 2006). Interestingly, Gcn4 and Stp1 have no apparent role in the transcriptional activation of *TPO1* gene induced by the lipophilic weak acid herbicide 2,4-D (results not shown) rendering clear that the regulatory network controlling *TPO1* transcription is largely dependent on the environmental stressor under study. Further studies are required to understand the physiological cues that determine which players of the *TPO1* regulatory network are activated in each condition.

Given the essential role played by Gcn4 and Stp1 in sensing and signalling internal amino acid homeostasis in yeast (Hinnebusch 2005; Ljungdahl and Daignan-Fornier 2012), the indications gathered prompted us to examine the effect of benzoic acid and *TPO1* transcription in the internal amino acid pool during early response to sudden exposure to this stress. However, *TPO1* deletion was found to have no detectable effect in the intracellular concentration of any of the measured amino acids in benzoic acid-supplemented media. These results suggest that, despite being a target of Gcn4/Stp1 regulatory control, Tpo1 is apparently not involved in the control of amino acid homeostasis in benzoic acid-stressed cells, a biological role that was proposed for its close homologues Aqr1 and Qdr2, also transcriptionally regulated by Gcn4 (Vargas et al. 2007; Velasco et al. 2004). Nonetheless, benzoic acid challenge was indeed seen to alter the intracellular amino acid pool in *S. cerevisiae* cells. In particular, the significant reduction in the internal concentration of glutamine, arginine and lysine (the latter reached undetectable levels) registered in benzoic acid-challenged cells could trigger activation of Gcn4 as this transcription factor responds when the internal concentration of any amino acid becomes limiting (Hinnebusch 2005). Intracellular acidification, a known deleterious effect of benzoic acid stress (Piper et al. 2001), was recently shown to reduce the activity of aminoacyl transfer RNA (tRNA) synthetases thereby leading to an accumulation of uncharged tRNAs (Hueso et al. 2012), a signal that is also known to activate Gcn4 (Hinnebusch 2005). The pool of the amino acids leucine and methionine that corresponds to the yeast strain auxotrophies did not suffer a reduction on the intracellular pools in benzoic acid-stressed cells; the levels of this amino acids were even found to increase. The increase in methionine and leucine intracellular levels in the same yeast cells challenged with propionic acid stress was previously registered by metabolomic analysis (Lourenço et al. 2011). The activation of the transcription factor Stp1 is dependent on the external sensing of amino acids by the receptor membrane protein Ssy1. It was proposed that when the concentration of an inducing amino acid in the exterior is higher than the concentration found in the cytosol, Ssy1 conformation is altered and Stp1 becomes active (Ljungdahl and Daignan-Fornier 2012). The activation of Stp1 registered under the experimental conditions used in our study was unexpected since a saturating concentration of ammonium was present in the growth medium, this being a condition that represses all pathways required for utilization of amino acids, including the Stp1 pathway (Ljungdahl and Daignan-Fornier 2012). It is possible that some of the amino acids whose internal concentration is reduced upon benzoic acid challenge could be accumulating in the exterior, as the result of either excretion and/or leakage, thereby resulting in the conformational change of Ssy1 and consequently in the activation of Stp1 as hypothesised before (Gaber et al. 2003; Ljungdahl and Daignan-Fornier 2012). Sudden exposure to benzoic acid stress was also found to lead to a reduction in the internal concentrations of spermidine, spermine and putrescine, this response being independent of the expression of the *TPO1* gene. This result appears to suggest that Tpo1 does not mediate the export of polyamines in response to benzoic acid stress. Altogether, the results of our study confirm that the transcriptional regulatory network that governs the expression of *TPO1* is complex and involves regulators that are not themselves directly implicated in MDR/MXR. Despite the strong Gcn4- and Stp1-dependent up-regulation of the *TPO1* gene registered under benzoic acid stress, the expression of Tpo1 did not have a significant effect in the internal amino acid pool in benzoic acid-stressed cells. Since the modulation of the internal concentration of polyamines was found to have a very pleiotropic effect in yeast cells (Chattopadhyay et al. 2008, 2009; Eisenberg et al. 2009), it is likely that the reduction in the internal concentration of polyamines could contribute in various manners to improve cell tolerance to benzoic acid, but this does not seem to be related with the role of the Tpo1 transporter in alleviating benzoic acid stress effects.

In conclusion, the results obtained in this study are expected to advance current understanding of the regulation and function of drug/xenobiotic efflux pumps in the MDR phenomenon in the yeast model and in less accessible organisms. Furthermore, since benzoic acid is largely used as a food preservative, it is expected that the identification of molecular mechanisms of tolerance to this weak acid in *S. cerevisiae* can be used to guide the design of more efficient preservation strategies, also at the level of medium composition in the food industry (Mira et al. 2010b). This is particularly expected considering that multiple robust homologues of Tpo1 are found in the genome sequence of several spoilage yeasts and fungi tolerant to benzoic acid, including strains of the food spoilage *Zygosaccharomyces bailii* species (Mira et al. 2014). Also, production of industrially relevant aromatic compounds using microorganisms has gathered increasing research interest. The production of benzoic acid through the assimilation of many carbon sources via a plant-like β-oxidation pathway was recently reported (Noda et al. 2012). Therefore, unveiling the mechanisms by which microbial strains are able to tolerate increasingly higher concentrations of this stress agent is also of extreme value to improve industrial robustness and reach enhanced production yield of benzoic acid-derived compounds.

## Electronic supplementary material

ESM 1 (PDF 314 kb)

## References

[CR1] Abbott DA, Knijnenburg TA, de Poorter LM, Reinders MJ, Pronk JT, van Maris AJ (2007). Generic and specific transcriptional responses to different weak organic acids in anaerobic chemostat cultures of *Saccharomyces cerevisiae*. FEMS Yeast Res.

[CR2] Albertsen M, Bellahn I, Kramer R, Waffenschmidt S (2003). Localization and function of the yeast multidrug transporter Tpo1p. J Biol Chem.

[CR3] Alenquer M, Tenreiro S, Sá-Correia I (2006). Adaptive response to the antimalarial drug artesunate in yeast involves Pdr1p/Pdr3p-mediated transcriptional activation of the resistance determinants *TPO1* and *PDR5*. FEMS Yeast Res.

[CR4] Aouida M, Rubio-Texeira M, Thevelein JM, Poulin R, Ramotar D (2013). Agp2, a member of the yeast amino acid permease family, positively regulates polyamine transport at the transcriptional level. PLoS One.

[CR5] Berra S, Ayachi S, Ramotar D (2014). Upregulation of the *Saccharomyces cerevisiae* efflux pump Tpo1 rescues an Imp2 transcription factor-deficient mutant from bleomycin toxicity. Environ Mol Mutagen.

[CR6] Borrull A, López-Martínez G, Poblet M, Cordero-Otero R, Rozès N (2015). New insights into the toxicity mechanism of octanoic and decanoic acids on *Saccharomyces cerevisiae*. Yeast.

[CR7] Cabrito TR, Teixeira MC, Duarte AA, Duque P, Sá-Correia I (2009). Heterologous expression of a Tpo1 homolog from *Arabidopsis thaliana* confers resistance to the herbicide 2,4-D and other chemical stresses in yeast. Appl Microbiol Biotechnol.

[CR8] Cabrito TR, Teixeira MC, Singh A, Prasad R, Sá-Correia I (2011). The yeast ABC transporter Pdr18 (ORF YNR070w) controls plasma membrane sterol composition, playing a role in multidrug resistance. Biochem J.

[CR9] Carrillo E, Ben-Ari G, Wildenhain J, Tyers M, Grammentz D, Lee TA (2012). Characterizing the roles of Met31 and Met32 in coordinating Met4-activated transcription in the absence of Met30. Mol Biol Cell.

[CR10] Chattopadhyay MK, Park MH, Tabor H (2008). Hypusine modification for growth is the major function of spermidine in *Saccharomyces cerevisiae* polyamine auxotrophs grown in limiting spermidine. Proc Natl Acad Sci U S A.

[CR11] Chattopadhyay MK, Chen W, Poy G, Cam M, Stiles D, Tabor H (2009). Microarray studies on the genes responsive to the addition of spermidine or spermine to a *Saccharomyces cerevisiae* spermidine synthase mutant. Yeast.

[CR12] Desmoucelles C, Pinson B, Saint-Marc C, Daignan-Fornier B (2002). Screening the yeast “disruptome” for mutants affecting resistance to the immunosuppressive drug, mycophenolic acid. J Biol Chem.

[CR13] Dias PJ, Teixeira MC, Telo JP, Sá-Correia I (2010). Insights into the mechanisms of toxicity and tolerance to the agricultural fungicide mancozeb in yeast, as suggested by a chemogenomic approach. OMICS.

[CR14] do Valle Matta MA, Jonniaux JL, Balzi E, Goffeau A, van den Hazel B (2001). Novel target genes of the yeast regulator Pdr1p: a contribution of the *TPO1* gene in resistance to quinidine and other drugs. Gene.

[CR15] Dos Santos SC, Sá-Correia I (2015). Yeast toxicogenomics: lessons from a eukaryotic cell model and cell factory. Curr Opin Biotech.

[CR16] Dos Santos SC, Teixeira MC, Dias PJ, Sá-Correia I (2014). MFS transporters required for multidrug/multixenobiotic (MD/MX) resistance in the model yeast: understanding their physiological function through post-genomic approaches. Front Physiol.

[CR17] Eisenberg T, Knauer H, Schauer A, Büttner S, Ruckenstuhl C, Carmona-Gutierrez D, Ring J, Schroeder S, Magnes C, Antonacci L, Fussi H, Deszcz L, Hartl R, Schraml E, Criollo A, Megalou E, Weiskopf D, Laun P, Heeren G, Breitenbach M, Grubeck-Loebenstein B, Herker E, Fahrenkrog B, Fröhlich K, Sinner F, Tavernarakis N, Minois N, Kroemer G, Madeo F (2009). Induction of autophagy by spermidine promotes longevity. Nat Cell Biol.

[CR18] Fernandes AR, Mira NP, Vargas RC, Canelhas I, Sá-Correia I (2005). *Saccharomyces cerevisiae* adaptation to weak acids involves the transcription factor Haa1p and Haa1p-regulated genes. Biochem Biophys Res Commun.

[CR19] Gaber RF, Ottow K, Andersen HA, Kielland-Brandt MC (2003). Constitutive and hyperresponsive signaling by mutant forms of *Saccharomyces cerevisiae* amino acid sensor Ssy1. Eukaryot Cell.

[CR20] Hill JE, Myers AM, Koerner TJ, Tzagoloff A (1986). Yeast/*E. coli* shuttle vectors with multiple unique restriction sites. Yeast.

[CR21] Hillenmeyer ME, Fung E, Wildenhain J, Pierce SE, Hoon S, Lee W, Proctor OR, Tyers M, Koller D, Altman RB, Davis RW, Nislow C, Giaever G (2008). The chemical genomic portrait of yeast: uncovering a phenotype for all genes. Science.

[CR22] Hinnebusch AG (2005). Translational regulation of *GCN4* and the general amino acid control of yeast. Annu Rev Microbiol.

[CR23] Hueso G, Aparicio-Sanchis R, Montesinos C, Lorenz S, Murguia JR, Serrano R (2012). A novel role for protein kinase Gcn2 in yeast tolerance to intracellular acid stress. Biochem J.

[CR24] Kitamoto K, Yoshizawa K, Ohsumi Y, Anraku Y (1988). Dynamic aspects of vacuolar and cytosolic amino acid pools of *Saccharomyces cerevisiae*. J Bacteriol.

[CR25] Klasson H, Fink GR, Ljungdahl PO (1999). Ssy1p and Ptr3p are plasma membrane components of a yeast system that senses extracellular amino acids. Mol Cell Biol.

[CR26] Krüger A, Vowinckel J, Mülleder M, Grote P, Capuano F, Bluemlein K, Ralser M (2013). Tpo1-mediated spermine and spermidine export controls cell cycle delay and times antioxidant protein expression during the oxidative stress response. EMBO Rep.

[CR27] Legras JL, Erny C, Le Jeune C, Lollier M, Adolphe Y, Demuyter C, Delobel P, Blondin B, Karst F (2010). Activation of two different resistance mechanisms in *Saccharomyces cerevisiae* upon exposure to octanoic and decanoic acids. Appl Environ Microbiol.

[CR28] Ljungdahl PO, Daignan-Fornier B (2012). Regulation of amino acid, nucleotide, and phosphate metabolism in *Saccharomyces cerevisiae*. Genetics.

[CR29] Lourenço A, Ascenso J, Sá-Correia I (2011). Metabolic insights into the yeast response to propionic acid based on high resolution 1H NMR spectroscopy. Metabolomics.

[CR30] Lucau-Danila A, Lelandais G, Kozovska Z, Tanty V, Delaveau T, Devaux F, Jacq C (2005). Early expression of yeast genes affected by chemical stress. Mol Cell Biol.

[CR31] Mima S, Ushijima H, Hwang HJ, Tsutsumi S, Makise M, Yamaguchi Y, Tsuchiya T, Mizushima H, Mizushima T (2007). Identification of the *TPO1* gene in yeast, and its human orthologue *TETRAN*, which cause resistance to NSAIDs. FEBS Lett.

[CR32] Mira NP, Lourenco AB, Fernandes AR, Becker JD, Sá-Correia I (2009). The RIM101 pathway has a role in *Saccharomyces cerevisiae* adaptive response and resistance to propionic acid and other weak acids. FEMS Yeast Res.

[CR33] Mira NP, Palma M, Guerreiro JF, Sá-Correia I (2010). Genome-wide identification of *Saccharomyces cerevisiae* genes required for tolerance to acetic acid. Microb Cell Factories.

[CR34] Mira NP, Teixeira MC, Sá-Correia I (2010). Adaptive response and tolerance to weak acids in *Saccharomyces cerevisiae*: a genome-wide view. OMICS.

[CR35] Mira NP, Becker JD, Sá-Correia I (2010). Genomic expression program involving the Haa1p-regulon in *Saccharomyces cerevisiae* response to acetic acid. OMICS.

[CR36] Mira NP, Münsterkötter M, Dias-Valada F, Santos J, Palma M, Roque FC, Guerreiro JF, Rodrigues F, Sousa MJ, Leão C, Güldener U, Sá-Correia I (2014). The genome sequence of the highly acetic acid-tolerant *Zygosaccharomyces bailii*-derived interspecies hybrid strain ISA1307, isolated from a sparkling wine plant. DNA Res.

[CR37] Moxley JF, Jewett MC, Antoniewicz MR, Villas-Boas SG, Alper H, Wheeler RT, Tong L, Hinnebusch AG, Ideker T, Nielsen J, Stephanopoulos G (2009). Linking high-resolution metabolic flux phenotypes and transcriptional regulation in yeast modulated by the global regulator Gcn4p. Proc Natl Acad Sci U S A.

[CR38] Myers AM, Tzagoloff A, Kinney DM, Lusty CJ (1986). Yeast shuttle and integrative vectors with multiple cloning sites suitable for construction of *lacZ* fusions. Gene.

[CR39] Noda S, Kitazono E, Tanaka T, Ogino C, Kondo A (2012). Benzoic acid fermentation from starch and cellulose via a plant-like beta-oxidation pathway in *Streptomyces maritimus*. Microb Cell Factories.

[CR40] Piper P, Calderon CO, Hatzixanthis K, Mollapour M (2001). Weak acid adaptation: the stress response that confers yeasts with resistance to organic acid food preservatives. Microbiology.

[CR41] Sá-Correia I, dos Santos SC, Teixeira MC, Cabrito TR, Mira NP (2009). Drug:H^+^ antiporters in chemical stress response in yeast. Trends Microbiol.

[CR42] Shimazu M, Sekito T, Akiyama K, Ohsumi Y, Kakinuma Y (2005). A family of basic amino acid transporters of the vacuolar membrane from *Saccharomyces cerevisiae*. J Biol Chem.

[CR43] Simões T, Teixeira MC, Fernandes AR, Sá-Correia I (2003). Adaptation of *Saccharomyces cerevisiae* to the herbicide 2,4-dichlorophenoxyacetic acid, mediated by Msn2p- and Msn4p-regulated genes: important role of SPI1. Appl Environ Microbiol.

[CR44] Smith JJ, Ramsey SA, Marelli M, Marzolf B, Hwang D, Saleem RA, Rachubinski RA, Aitchison JD (2007). Transcriptional responses to fatty acid are coordinated by combinatorial control. Mol Syst Biol.

[CR45] Tang L, Liu X, Clarke ND (2006). Inferring direct regulatory targets from expression and genome location analyses: a comparison of transcription factor deletion and overexpression. BMC Genomics.

[CR46] Teixeira MC, Sá-Correia I (2002). *Saccharomyces cerevisiae* resistance to chlorinated phenoxyacetic acid herbicides involves Pdr1p-mediated transcriptional activation of *TPO1* and *PDR5* genes. Biochem Biophys Res Commun.

[CR47] Teixeira MC, Fernandes AR, Mira NP, Becker JD, Sá-Correia I (2006). Early transcriptional response of *Saccharomyces cerevisiae* to stress imposed by the herbicide 2,4-dichlorophenoxyacetic acid. FEMS Yeast Res.

[CR48] Teixeira MC, Monteiro P, Jain P, Tenreiro S, Fernandes AR, Mira NP, Alenquer M, Freitas AT, Oliveira AL, Sá-Correia I (2006). The YEASTRACT database: a tool for the analysis of transcription regulatory associations in *Saccharomyces cerevisiae*. Nucl Acids Res.

[CR49] Teixeira MC, Duque P, Sá-Correia I (2007). Environmental genomics: mechanistic insights into toxicity of and resistance to the herbicide 2,4-D. Trends Biotechnol.

[CR50] Teixeira MC, Cabrito TR, Hanif ZM, Vargas RC, Tenreiro S, Sá-Correia I (2011). Yeast response and tolerance to polyamine toxicity involving the drug:H^+^ antiporter Qdr3 and the transcription factors Yap1 and Gcn4. Microbiology.

[CR51] Teixeira MC, Mira NP, Sá-Correia I (2011). A genome-wide perspective on the response and tolerance to food-relevant stresses in *Saccharomyces cerevisiae*. Curr Opin Biotech.

[CR52] Teixeira MC, Monteiro PT, Guerreiro JF, Gonçalves JP, Mira NP, dos Santos SC, Cabrito TR, Palma M, Costa C, Francisco AP, Madeira SC, Oliveira AL, Freitas AT, Sá-Correia I (2014). The YEASTRACT database: an upgraded information system for the analysis of gene and genomic transcription regulation in *Saccharomyces cerevisiae*. Nucl Acids Res.

[CR53] Thakur JK, Arthanari H, Yang F, Pan SJ, Fan X, Breger J, Frueh DP, Gulshan K, Li DK, Mylonakis E, Struhl K, Moye-Rowley WS, Cormack BP, Wagner G, Näär AM (2008). A nuclear receptor-like pathway regulating multidrug resistance in fungi. Nature.

[CR54] Tomitori H, Kashiwagi K, Asakawa T, Kakinuma Y, Michael AJ, Igarashi K (2001). Multiple polyamine transport systems on the vacuolar membrane in yeast. Biochem J.

[CR55] Uemura T, Tachihara K, Tomitori H, Kashiwagi K, Igarashi K (2005). Characteristics of the polyamine transporter *TPO1* and regulation of its activity and cellular localization by phosphorylation. J Biol Chem.

[CR56] Uemura T, Kashiwagi K, Igarashi K (2007). Polyamine uptake by DUR3 and SAM3 in *Saccharomyces cerevisiae*. J Biol Chem.

[CR57] Vargas RC, Garcia-Salcedo R, Tenreiro S, Teixeira MC, Fernandes AR, Ramos J, Sá-Correia I (2007). *Saccharomyces cerevisiae* multidrug resistance transporter Qdr2 is implicated in potassium uptake, providing a physiological advantage to quinidine-stressed cells. Eukaryot Cell.

[CR58] Velasco I, Tenreiro S, Calderon IL, André B (2004). *Saccharomyces cerevisiae* Aqr1 is an internal-membrane transporter involved in excretion of amino acids. Eukaryot Cell.

[CR59] Viegas CA, Cabral MG, Teixeira MC, Neumann G, Heipieper HJ, Sá-Correia I (2005). Yeast adaptation to 2,4-dichlorophenoxyacetic acid involves increased membrane fatty acid saturation degree and decreased *OLE1* transcription. Biochem Biophys Res Commun.

