# Supplementary material for: Yeast response and tolerance to benzoic acid involves the Gcn4- and Stp1-regulated multidrug/multixenobiotic resistance transporter Tpo1
Source: Appl Microbiol Biotechnol. 2017 Apr 13;101(12):5005–18. doi: 10.1007/s00253-017-8277-6 (PMC5486834; doi:10.1007/s00253-017-8277-6)
Supplement: Supplementary file 1 — (PDF 314 kb) [file 253_2017_8277_MOESM1_ESM.pdf]

Applied Microbiology and Biotechnology

Yeast response and tolerance to benzoic acid involves the Gcn4- and Stp1-regulated multidrug/multixenobiotic resistance transporter Tpo1

Cláudia P. Godinho[1], Nuno P. Mira[1], Tânia R. Cabrito[1], Miguel C. Teixeira[1], Kaur Alasoo[1], Joana F. Guerreiro[1] and Isabel Sá-Correia[1]✦

[1]iBB, Institute for Bioengineering and Biosciences, Department of Bioengineering, Instituto Superior Técnico, Universidade de Lisboa, Avenida Rovisco Pais, 1049-001 Lisbon, Portugal;

✦corresponding author: Prof. Isabel Sá-Correia, Ciências Biológicas Instituto Superior Técnico, Av. Rovisco Pais, 1049-001 Lisboa, Portugal; e-mail: isacorreia@tecnico.ulisboa.pt; Tel. +351-218417682;

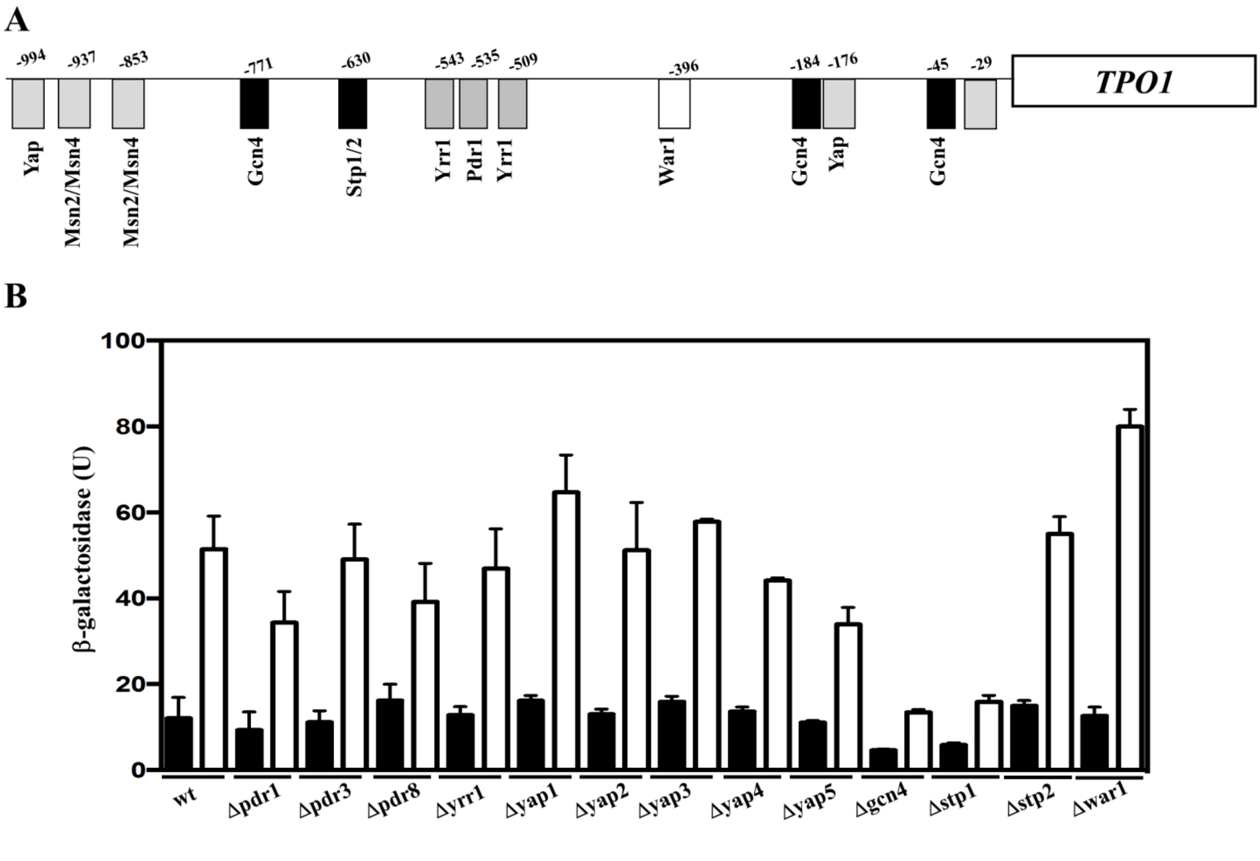

Supplementary Fig. S1 (A) Schematic representation of the DNA motifs existing in the *TPO1* promoter region that serve as binding sites for the stress-responsive transcription factors Pdr1, Pdr3, Yrr1, Stp1, Msn2, Msn4, Yap1, Yap2, Yap3, Yap4, Yap5 and Gcn4; (B) Expression of *TPO1* gene, assessed based on the levels of β-galactosidase activity produced from the plasmid p*TPO1*::*lacZ* (Alenquer et al. 2006), in wild-type and in mutants deleted for the above referred transcription factors after 12 h of growth in MM4 growth medium (at pH 4) (black bars) or in this same growth medium supplemented with 0.9 mM benzoic acid (white bars). The results presented are means of at least three independent experiments.
